# Dependences of Q-branch integrated intensity of linear-molecule pendular spectra on electric-field strength and rotational temperature and its potential applications

**DOI:** 10.1038/srep26776

**Published:** 2016-05-27

**Authors:** Min Deng, Hailing Wang, Qin Wang, Jianping Yin

**Affiliations:** 1 State Key Laboratory of Precision Spectroscopy, Department of Physics, East China Normal University, Shanghai 200062, P. R. China

## Abstract

We calculate the pendular-state spectra of cold linear molecules, and investigated the dependences of “Q-branch” integrated intensity of pendular spectra on both electric-field strength and molecular rotation-temperature. A new multi-peak structure in the “Q-branch” spectrum is appearing when the Stark interaction strength *ω* = *μE/B* equal to or larger than the critical value. Our study shows that the above results can be used not only to measure the electric-field vector and its spatial distribution in some electrostatic devices, such as the Stark decelerator, Stark velocity filter and electrostatic trap and so on, but also to survey the orientation degree of cold linear molecules in a strong electrostatic field.

Since a new concept on pendular states of molecules in strong electric field was first proposed in 1991^1^ and the pendular-state spectra of (HCN)_3_ in strong electric fields were first reported in 1992^2^,^3^, theoretical^4^,^5^,^6^,^7^,^8^ and experimental^9^,^10^,^11^,^12^,^13^,^14^ investigations on the pendular-state properties and their spectrum structures of cold polar molecules have made a great progress. These studies show that such a novel pendular-state spectrum can be used to precisely measure molecular electric dipole moment^15^,^16^ and its change^17^, to study some new complexes, such as, NH_3_CN complex^18^ and CH_3_---H_2_O radical complex^19^, and so on. In addition, oriented cold/ultracold polar molecules have important applications in quantum information science^20^, biochemistry, and biophysics^21^.

However, how to prepare cold/ultracold molecules is a very important scientific question and has become one of hot points in the fields of atomic, molecular and optical physics (AMO), quantum dipole gases and quantum information science. cold/ultracold molecules attract great interests of scientists to prepare and study them because they have many important applications in cold collisions and cold chemistry, molecular BEC (Bose-Einstein condensate) and Fermi quantum degeneration, quantum and nonlinear molecule optics, cold molecular spectroscopy and precise measurement, integrated molecule optics and molecule chip, quantum computing and information processing, and so on. Particularly, cold/ultracold polar molecules can be used to study their quantum-state controlled pendular spectrum. Currently, they can be prepared and manipulated by using some electrostatic devices, such as Stark decelerator^22^ and velocity filter^23^,^24^, electrostatic guide^25^ and beam splitter^26^, electrostatic trap^27^, and laser cooling^28^,^29^, etc.

The accurate amplitude and direction of the electrostatic fields and their spatial distributions play important roles in the preparation and investigation of cold/ultracold molecules using above electrostatic devices^22^,^23^,^24^,^25^,^26^,^27^. The electric field (E-field) strength in these electrostatic devices^22^,^23^,^24^,^25^,^26^,^27^ is varied from 0 to 100 kV cm^−1^. Also, the measurements of externally-added the electrostatic field vector and its spatial gradient as well as the orientation degree (i.e., the polarizability) of cold heavy-atom molecules (such as YbF^30^, PbF^31^, PbO^32^ and ThO^33^, etc.) in the strong electric field play an important role in precise measurement of electron electric polar momentum (eEDM)^30^,^31^,^32^,^33^. It is well known that the E-field vector and its spatial distribution are basic and important physical quantities, and their measurement to date have not been solved well, unlike the measurement of the magnetic field^34^. In recent years, only a few methods have been developed to measure the E-field amplitude or the E-field vector and its spatial distribution from a weak E-field (∼1 V cm^−1^) to a moderate one (∼10^3 ^V cm^−1^) by using electro-optic probe^35^, Stark spectrum of Rydberg-atoms^36^,^37^ and Rydberg-molecules^38^, and fluorescence-dip spectroscopy^39^ and so on^40^,^41^. Those methods only cover a narrow range of around three orders of magnitude which is far narrower than the measuring range of the magnetic field from 10^−15^ T to 10^3^ T^34^. However, the above measurement methods^35^,^36^,^37^,^38^,^39^,^40^,^41^ cannot be used to measure *in situ* the E-field vector and its spatial distribution in the electrostatic devices^22^,^23^,^24^,^25^,^26^,^27^ and eEDM experiments^30^,^31^,^32^,^33^. So it would be interesting and worthwhile to investigate the dependences of Q-branch intensity of pendular-state spectrum of cold/ultracold linear molecules on both the E-field strength and the molecular rotational temperature, and then develop a new and desirable method to measure the E-field vector and its spatial distribution with an ultra-wide surveying range. In this paper, we first present a method to calculate the pendular-state spectra of cold/ultracold linear molecules, and then study the dependences of Q-branch integrated intensity of pendular-state spectra of cold/ultracold linear molecules on both the E-field strength and the molecular rotational temperature (MRT). Also, we discuss some potential applications of the cold/ultracold linear molecule pendular-state spectrum in the measurement of the E-field vector and its spatial distribution as well as the orientation degree (i.e., the polarizability) of cold molecules. The main results and conclusions are included in final section.

## Results

### Calculation of pendular-state spectrum

**Figure 1:**
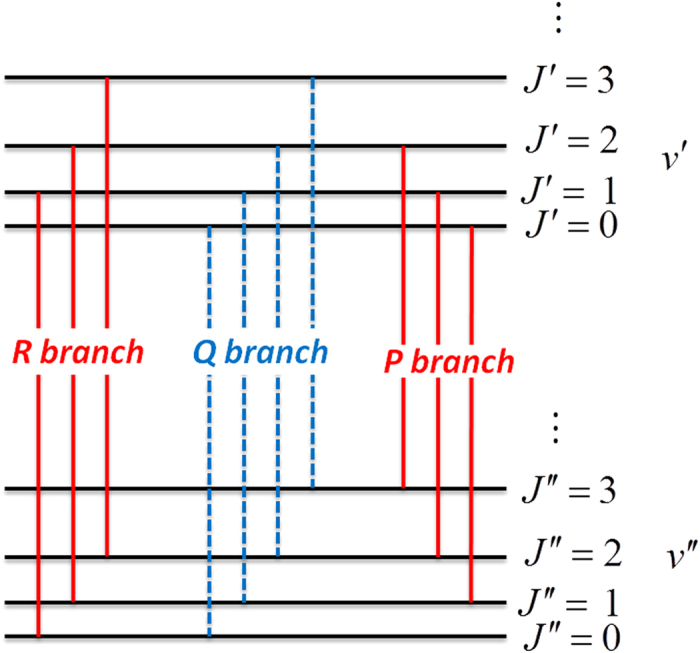
Energy level diagram of the pure-rovibrational band of linear (HCCCN)_3_ with P-, R-branches (there is no Q-branch according to the selection rule).

Figure 1 shows the energy level diagram of the pure-rovibrational band of linear molecules (HCCCN)_3_ with P-, R-branches. The Q-branch transition of (HCCCN)_3_ is forbidden due to the selection rule. However, a so called “Q-branch” transition of the linear (HCCCN)_3_ will appearing in an external electric field due to the mixture of the wavefunctions (i.e., due to the appearance of pendular state). Generally, polar molecules in a strong electric field will be oriented and converted from pinwheeling rotors into pendular liberators confined to oscillate over a limited angular range along the field direction. In this case, the corresponding rotation eigenstates become the pendular states. Molecules with a low rotational temperature, T, a large dipole moment, and a small molecular constant, B, are very easy to be implemented into the pendular state under a weak electric field. The pendular-state spectra were first investigated experimentally by Miller’s group^3^.

The pendular states are linear combinations of the field-free rotor states, 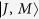, where J is the rotational quantum number, M is the projection of the angular momentum J along the direction of the E-filed vector^2^,^3^. For linear molecules with an electric dipole moment and a rotational constant B in an E-field, the ratio *ω* = *μE*(*r*)/*B* was used to govern the extent of hybridization of the rotor states. The Schrödinger equation of a rigid linear molecule in an E-field is given by


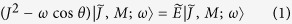


where *J*^2^ is the squared angular momentum operator, *M* is the projection of the angular momentum *J* along the E-field vector direction, and *θ* is the angle between the molecular axis and the E-field vector, and 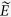 is the energy eigenvalue. The Stark eigenstate is labeled by the rotational state of the field-free case, and given by


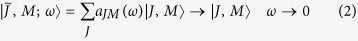


where *a*_*JM*_(*ω*) is the coefficient, which specifies the eigenfunction as linear combination of all rotational levels in a vibrational state. For any fixed value of the good quantum number *M*, the range of *J* involved in this coherent superposition or hybrid wavefunction increases with the ω parameter. The eigenstates are labeled by *M* and the nominal value of 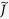 of the angular momentum for the field-free rotor state that adiabatically correlates with the high-field hybrid function. For ω > 0, the states with different values of |M| within each 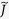 manifold have different energies^42^. For transitions between a pair of pendular states, 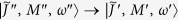, and the line-strength factor is given by^42^


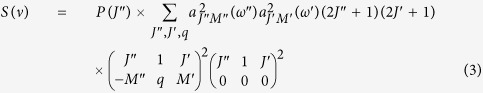


And the corresponding normalized integrated intensity of the Q-branch spectra is given by


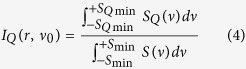


where *v* is the vibratioanl transition frequency; 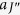 and 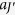 are the coefficients introduced by the field-induced hybridization of *J*′ and *J*″ states give rise to non-zero transition probabilities between the states that differ by more than unity in their nominal 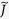 value; 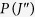 is the relative population of initial thermal equilibrium, which meets the Maxwell-Boltzmann distribution. In our calculations, the redistribution process of molecules in the E-field was assumed as an adiabatic one, and thus 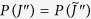. *q* (=*0,* ±*1*) designates the spherical components in the space-fixed frame of the E-field vector of the radiation. q = 0 means that the polarization of the probe beam is parallel to the direction of the E-field vector; q = ±1 means that these two directions are orthogonal each other. The relative strength *I*_*Q*_(*r*,* v*_0_) of the “Q-branch” can be calculated by using Eq. (4), here v_0_ is the band origin. ±S_Qmin_ and ±S_min_ are the minimum values of the “Q-branch” and entire ro-vibrational spectra’s both sides. The spectrum line profile was considered as Gaussian function, its full width at half maximum (FWHM) was set as B/4^43^.

**Figure 2:**
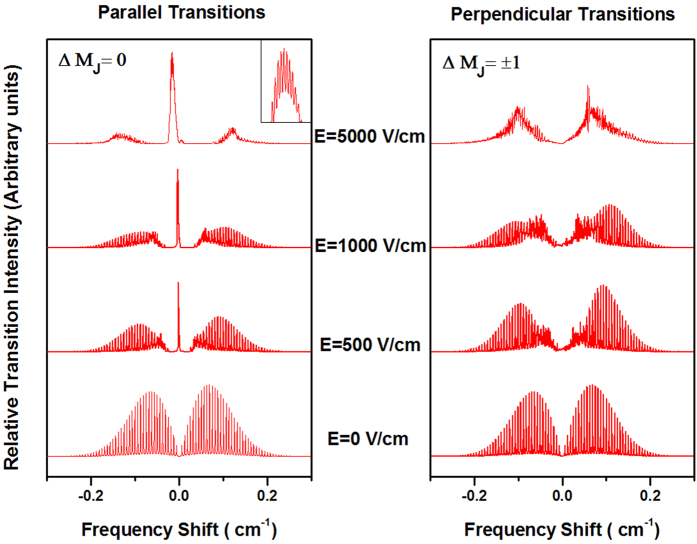
Calculated rotationally resolved spectrum of (HCCCN)_3_ molecule based on the molecular constants listed in Table 1. The lower abscissa scale is in cm^−1^ relative to the band origin of the vibrational mode of C-H stretch.

The spectra of possible rovibrational transitions of the *v*_1_ vibrational mode of C-H stretch of linear cyanoacetylene trimer (HCCCN)_3_^43^ in various applied E-field strengths (including *E* = 0) were calculated by using Eq. (3), and the results are shown in Fig. 2. Table 1 shows the three kinds of molecular parameters in our calculation. It is clear that our calculated parallel transition spectrum (see the left spectrum in Fig. 2) of (HCCCN)_3_ molecule are similar to the experimental ones of (HCN)_3_ 3, HCCCN^16^, (HCCCN)_3_ 18, and C_2_H_4_ 44. This shows that these experimental spectrums verify our calculated results (see Fig. 2).

We can see from Fig. 2 that there are only P- and R-branch transitions that are allowed in free external field, the Q-branch is forbidden [see Fig. 1]. However, the mixing of the wave functions of the linear molecules in the electric fields will lead to the appearance of so-called “Q-branch” forbidden lines in the laser induced fluorescence (LIF) spectra, and the intensity of the new coming “Q-branch” depends on the E-field strength. The calculated rotationally resolved spectra of (HCCCN)_3_ under the parallel transition (the transitions of Δ*M*_*J*_ = 0) and the perpendicular transition (the transitions of Δ*M*_*J*_ = ±1) are showed in Fig. 2.

Figure 2 shows the change of the parallel and perpendicular transition rotational resolved spectra of (HCCN)_3_ in different electric fields. In the left side of Fig. 2, a “Q-branch” is appear and its intensity rise with the increase of the E-field strength. The higher the E-field strength is, the stronger the “Q-branch” intensity will be, and the weaker the P-, R-branch intensities will be, and this process is going faster under a lower molecular rotational temperature (MRT). This shows that cold/ultracold molecules are more sensitive samples for measuring the strength of weak E-fields. In particular, we find that when the E-field is high enough, the P- and R-branch spectra will be nearly disappear, while there is only a single Q-branch spectrum. In Fig. 2, a multi-peak spectral structure appears in the Q-branch when the Stark interaction strength 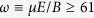 for (HCCCN)_3_ with a MRT of 1 K and rise with increasing the E-field strength, which is due to the slight difference of the electric dipole moments, *μ*, and molecular constants B of the upper and lower vibrational ground states, v and v″. To our knowledge, this multi-peak spectral structure is the first finding in the world. It is clear that such a multi-peak spectral structure can be used to precisely measure the electric dipole moments *μ* and *B* constants of the selected molecules. In the right side of Fig. 2, the P- and Q-branches show complicated structures and Stark shifts, and then they will finally collapse into two broad clumps with the increase of the E-field strength.

### Q-branch integrated intensity of pendular-state spectrum

**Figure 3:**
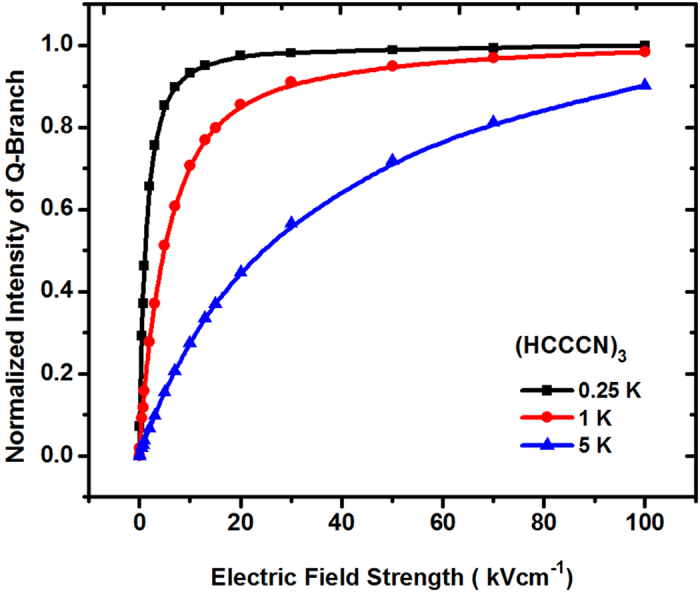
The dependences of the normalized Q-branch intensity on the E-field strength for different rotational temperature of (HCCCN)_3_.

The integrated intensity of the Q-branch of (HCCCN)_3_ under different E-field strength were calculated, and the dependence of the normalized intensity of the “Q-branch” of (HCCCN)_3_ with different MRT on the E-field strength E was studied by using Eq. (4), and the results are shown in Fig. 3. From Fig. 3 we can see that with the increase of E-field strength, the integrated intensity of the Q-branch will be increased quickly, then raised slowly, and reached to a saturated value finally. Due to static Stark shift, higher rotational levels will be mixed in stronger E-field easily. The intensity of the Q-branch reaches its saturated value when all rotational levels were mixed. Moreover, when the E-field strength is increased from 0 to 10 kV cm^−1^, the intensity of the “Q-branch” of (HCCCN)_3_ with a MRT of 0.25 K will be increased faster than that with a MRT of 5 K, and the low-energy rotational levels will be easily mixed in the E-field. This is because most of cold molecules are populated in lower rotational levels dominantly, thus the colder molecules will reach its saturation in the external E-field quickly, and has stronger relative intensity factor of Q-branch compared with that of a higher molecular temperature. In particular, when T = 0.25 K, the Q-branch integrated intensity of cold (HCCCN)_3_ will be increased from 0 to about 0.95 when the E-field strength is increased from 0 to 10 kV cm^−1^; while T = 5 K, it will be increased from 0 to ~0.90 with increasing E-field strength from 0 to 100 kV cm^−1^.

**Figure 4:**
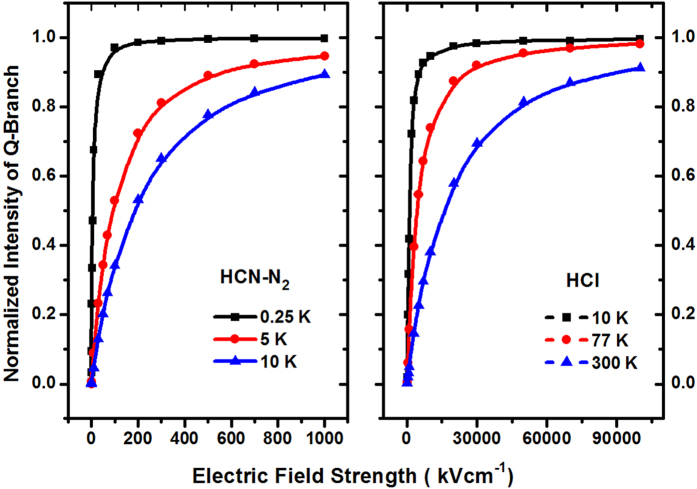
The dependences of the normalized Q-branch intensity of (left) HCN-N_2_ and (right) HCl molecules on the E-field strength for different rotational temperature.

We also investigate the dependence of the normalized integrated intensity of the Q-branch on the E-field strength for HCN-N_2_ 45 and HCl^42^, and the results are shown in Fig. 4. It is clear from Fig. 4(a) that when T = 5 K, the Q-branch integrated intensity of cold HCN-N_2_ will be increased from about 0.3 to 0.95 when the E-field strength is increased from 50 kV cm^−1^ to 1000 kV cm^−1^; while T = 10 K, it will be increased from about 0.35 to 0.90 with increasing the E-field strength from 100 kV cm^−1^ to 1000 kV cm^−1^. Also, we can see from Fig. 4(b) that when T = 77 K (i.e., the liquid nitrogen (LN) temperature), the Q-branch integrated intensity of cold HCN-N_2_ will be increased from about 0.35 to 0.98 when the E-field strength is increased from 5000 kV cm^−1^ to 10^5^ kV cm^−1^; while T = 300 K (i.e., the room temperature), it will be increased from about 0.3 to 0.92 with the increase of the E-field strength from 7500 kV cm^−1^ to 10^5^ kV cm^−1^. These results show that it is suitable to observe the Q-branch spectrum of cold linear (HCCCN)_3_, HCN-N_2_ and the room temperature HCl in a weak, moderate and strong E-field, respectively.

**Figure 5:**
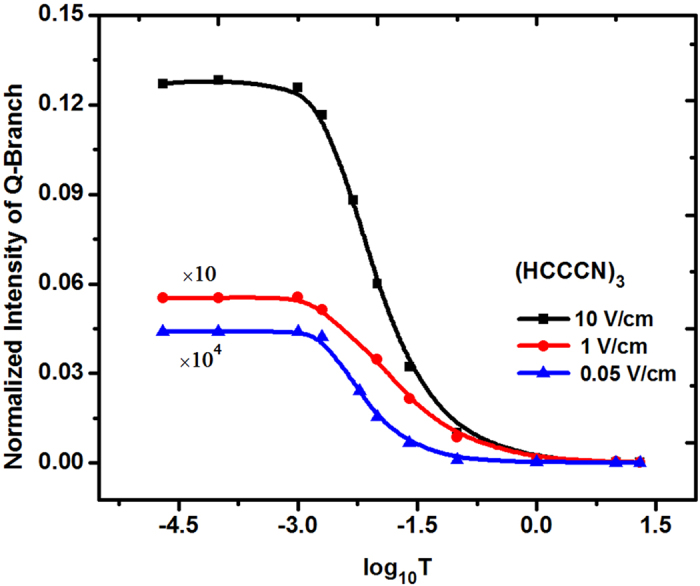
The dependences of the normalized Q-branch intensity on the rotational temperature of (HCCCN)_3_ for different E-field strength. In which, the Y-coordinate values of the red and blue lines are multiplied by 10 times and 10000 times in order to compare with the black one; the line width of each possible transition is changed by 0.00001 cm^−1^ (≈B/300) to obtain higher resolution under 0.05 V cm^−1^.

Figure 5 is the dependence of the normalized integrated intensity of (HCCCN)_3_ Q-branch on the MRT T, and shows that for a certain E-field strength, with the reduction of the MRT, the intensity of Q-branch will be first increased and then slowly reached to a saturation value. Particularly, when T ≤ 1 mK, the E-field measurement value of ultracold (HCCCN)_3_ can reach ∼ 0.01 V cm^−1^, even a smaller value. This is because the lower the MRT T is, the higher the population of lower rotational levels will be, and when the MRT T is low enough, the population of the lowest rotational level will reach its highest value, and then the integrated intensity of the Q-branch will reach its saturation value. This result shows that the lower the MRT is, the weaker the needed E-field will be, and ultracold linear molecules in a weaker E-field can be used to obtain a higher sensitivity for the observation of the Q-branch integrated intensity.

## Discussion

### Some potential applications

First, we can see from Fig. 2 that a newly-found multi-peak spectral structure can be used to precisely measure the electric dipole moments *μ* and *B* constants of different vibration states of cold linear molecules.

Secondly, it is clear from Figs 3, 4 and 5 that ultracold or cold (HCCCN)_3_ with a temperature of lower or higher than 1 mK is suitable to measure a weaker E-field strength within 10^−5^ ∼ 100 kV cm^−1^, cold HCN-N_2_ and heat HCl from 77 K to 300 K are suitable to measure a moderate E-field strength (1 ∼ 10^3^ kV cm^−1^) and a strong one (10^2^ ∼ 10^5^ kV cm^−1^) by using the pendular Q-branch spectra of the linear molecule, respectively. The measurement principle and method will be introduced in some detail as follows: in our proposed scheme, the space of the measured E-field will be filled with the selected cold linear molecule gas, which can be generated by using buffer-gas cooling and Stark velocity filter^23^,^24^ or Stark decelerator^22^ in a high vacuum chamber. A tunable single-mode, linear-polarized probe beam from an OPO/OPA laser system is focused into the chamber and used to excite cold molecule sample so as to generate the fluorescence spectra of the rovibrational transitions, and the resulting LIF spectra will be collected and detected by an IR detector system. If the fluorescence collective coefficient *η*_*coll*_, and the quantum efficiency *η*_*Det*_ of the detector were assumed as a constant near the band origin *v*_0_, the photoelectric current of the detected fluorescence intensity of the “Q-branch” can be given by


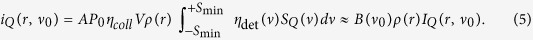


where *B*(*v*_0_) = *AP*_0_*η*_*coll*_*η*_det_, *A* is the system coefficient, *P*_0_ and *V* are the power of the probe beam and the effective probing volume, respectively. From Figs 3, 4 and 5, the measured E-field strength can be expressed as


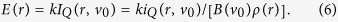


where *k* is the proportional coefficient, which can be determined by the Q-branch intensity of cold linear molecules in a homogeneous E-field generated by a pair of plate electrodes, that is, it can be determined by the calibration curve of the resulting Q-branch intensity of a plate capacitor. *ρ*(*r*) is the spatial density distribution of cold molecules and will be easily affected by the E-field environment, but this problem can be solved by using a CCD camera to obtain the fluorescent intensity distribution from the molecule sample, which is proportional to the spatial density distribution of cold molecules in an entire measurement region. This is similar to the measurement method of cold atomic density used in the MOT or BEC experiments.

**Figure 6:**
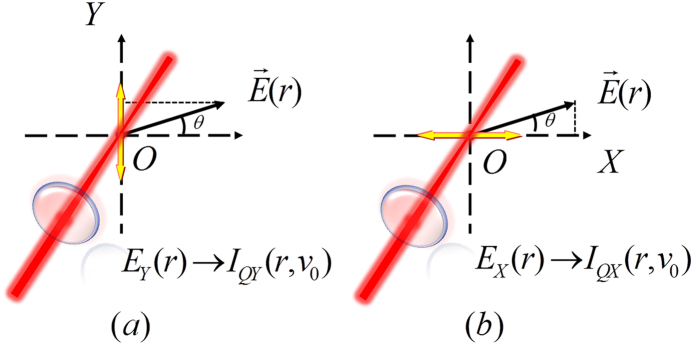
Schematic diagrams to measure (**a**) the Y component, *E*_*Y*_(*r*), and (**b**) the X component, *E*_*X*_(*r*), of the E-field vector. Yellow double arrow represents the polarization direction of the probe beam.

The basic principle for measuring the direction of the E-field vector can be simply described as follows: the magnitude and direction of the E-field vector in a two-dimensional (2D) plane (such as in XOY plane) can be measured by changing the direction of the probe-beam polarization two times. That is, from Eq. (6), when the direction of the probe-beam polarization is in the X direction, as shown in Fig. 6(a), the X component *E*_X_ of the measured E-field vector can be obtained by measuring the photon current *i*_QX_ from the corresponding LIF intensity *I*_QX_ of the Q-branch. If the direction of the probe-beam polarization is changed as the Y direction [see Fig. 6(b)], the Y component *E*_Y_ of the measured E-field vector will be obtained by measuring iQY from the corresponding *I*_QY_. Then, the magnitude 
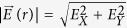
 of the measured E-field vector 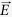 in 2D space and its azimuthal angle 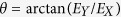 or 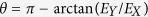 will be determined. Similarly, if the propagation direction of the probe beam is changed from the Z direction to the X or Y direction, and the direction of the laser polarization is changed to the Z direction, the Z component *E*_Z_ of the measured E-field vector can be determined by using similar measurement method, and then we can obtain the E-field vector 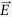 in 3D space.

Finally, it is well known that the stronger the electric field is, the higher the orientation degree of cold linear-molecules will be^1^, so the dependence of Q-branch integrated intensity on the electric-field strength can also be used to measure the orientation degree of cold linear-molecules.

### Measurement sensitivity

Similar to the derivation of measurement sensitivity expression in refs 46,47, when neglecting the inference of stray light on the measured LIF signal, the single-shot limited sensitivity of our method to measure the static electric field, determined by only shot noise resulting from the fluctuation of the fluorescence signal, is given by (see Supplementary material for more details)


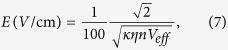


where *η*_*c*_ is the fluorescence collecting efficiency, *n* is the density of a supersonic molecular beam, and *κ* the front factor of *E*^2^. The effective detected volume within probe laser beam can be assumed as a spheroid, it’s volume *V*_*eff*_ equals to 
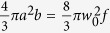
, here *w*_0_ is the waist radius, and *f* represents the Rayleigh length of the Gaussian beam. When the fluorescence collecting efficiency of detection system is *η*_*c*_ = 10%, the rotational temperature of a supersonic (HCCCN)_3_ beam is 5 K, and the waist radius of the focused probe beam is *w*_0_ = 10 μm, we obtain a static electric-field sensitivity of 2.05 × 10^−7^ V/cm^48^. It is clear that when SNR = 1, the relative measurement error (1/SNR, or measurement accuracy) reaches 100%. So in order to obtain a 1% relative measurement error (a relative uncertainty), the SNR should be equal to 100, and the corresponding practical sensitivity is E = 2.05 × 10^−5 ^V/cm, which is far lower than ones (5 V/cm ~ 100 V/cm) of other methods^36^,^38^,^39^.

## Conclusion

We have calculated pendular-state spectra of cold linear molecules, and studied the dependences of the integrated intensity of the Q-branch on both the E-field strength and the MRT of cold linear molecules, and found that these dependences can be used to sensitively measure the E-field vector and its spatial distribution by changing the polarization directions of the focused probe-beam as well as the orientation degree of cold linear molecules. Also, we have found some new physical phenomena as follows: (1) the saturation effect of the integrated intensity of the Q-branch in a strong E-field; (2) the similar saturation effect occurred at an ultralow temperature of the linear molecules, and (3) a multi-peak structure in the Q-branch spectrum as well as its appearing condition 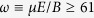 for (HCCCN)_3_, and given reasonable physical explanations. Our study shows that the proposed measurement method can cover an ultrawide range of about 10^−5^ V cm^−1^ ~ 10^5^ kV cm^−1^ theoretically, and the corresponding practical measurement sensitivity is 2.05 10^−5^ V cm^−1^. So it is clear that such a high-sensitive and ultrawide-range method can be used to realize the nonintrusive, *in-situ* measurement of the E-field vector and its spatial distribution in Stark decelerator^22^, velocity filter^23^,^24^, electrostatic guide^25^ and beam splitter^26^, and electrostatic trap^27^, and so on [49]. Also, it can be used to measure the E-field vector and its spatial gradient in eEDM experiment using heavy-atom molecules (such as YbF, PbF and ThO, etc.)^30^,^31^,^32^,^33^ because they have a larger EDM and a smaller B constant.

## Additional Information

**How to cite this article**: Deng, M. *et al*. Dependences of Q-branch integrated intensity of linear-molecule pendular spectra on electric-field strength and rotational temperature and its potential applications. *Sci. Rep.* **6**, 26776; doi: 10.1038/srep26776 (2016).

## Supplementary Material

Supplementary Information

## Figures and Tables

**Table 1:** Molecular parameters (***μ***, **B**) and values of *ω* = *μE*/*B* as E = 1 kV cm^−1^.

**Molecule**	***μ***^″^(*D*)	***μ***′(*D*)	***B***″**(cm**^**−1**^)	***B***′**(cm**^**−1**^)	***ω***	***v***_**0**_**(cm**^**−1**^)
(HCCCN)_3_ 43	11.4	11.67	0.00313654	0.00313626	61.03	3323.68245
HCN-N_2_ 45	3.04	3.085	0.052513	0.052138	0.972	3301.9389
HCl^42^	1.1085	1.139	10.44025	10.13623	0.00178	2885.9765
