# Supplementary material for: Dependences of Q-branch integrated intensity of linear-molecule pendular spectra on electric-field strength and rotational temperature and its potential applications
Source: Sci Rep. 2016 May 27;6:26776. doi: 10.1038/srep26776 (PMC4882541; doi:10.1038/srep26776)
Supplement: Supplementary Information [file srep26776-s1.doc]

Supplementary Information: Dependences of Q-branch integrated intensity of linear-molecule pendular spectra on electric-field strength and rotational temperature and its potential applications

Min Deng, Hailing Wang, Qin Wang and Jianping Yin*

State Key Laboratory of Precision Spectroscopy, Department of Physics, East China Normal University, Shanghai 200062, P. R. Chin

[*jpyin@phy.ecnu.edu.cn](mailto:*jpyin@phy.ecnu.edu.cn)

1. **The relationship between electric field and normalized Q-branch intensity in the weak field limit.**

In order to derive an equation to describe limit sensitivity of electric field measurement, we only need to consider the question of weak electric field measurement **(**$E\leq1 \text{V/cm}$**)**. In this case, a quadratic relationship between the electric field and normalized Q-branch intensity can be derived from perturbation theory in quantum mechanics.

## For a rotational state $|J,M\rangle$ in static electric field, the corresponding Stark eigenstate can be given by the [first order perturbation theory](http://www.docin.com/p-737800511.html),

## $\begin{matrix} |\tilde{J},M\rangle=|J,M\rangle+\frac{H'_{J,J-1}}{E_{J,M}-E_{J-1,M}}|J-1,M\rangle+\frac{H'_{J,J+1}}{E_{J,M}-E_{J+1,M}}|J+1,M\rangle\\ H'_{J,J-1}=-\langle J,M|\vec{p}\cdot\vec{E}_{s}|J-1,M\rangle,H'_{J,J+1}=-\langle J,M|\vec{p}\cdot\vec{E}_{s}|J+1,M\rangle\end{matrix}$. (1)

## The normalized Q-branch intensity $I_{Q}(v_{0},J')$ is proportional to

## $P(J') \left|\langle\tilde{J}',M|\vec{p}\cdot\vec{E}_{L}|\tilde{J}'',M\rangle\delta_{J',J''}\right|^{2}$, (2)

## where $\vec{p}$is the [electric](javascript:void(0);) [dipole](javascript:void(0);) [moment](javascript:void(0);) and$P(J')$ is the relative population of cold molecules in initial thermal equilibrium, which meets the Maxwell-Boltzmann distribution.

## $\begin{matrix} \langle\tilde{J}',M|\vec{p}\cdot\vec{E}_{L}|\tilde{J}'',M\rangle\delta_{J',J''} \\ =c'_{-}\langle J'-1,M|\vec{p}\cdot\vec{E}_{L}|J',M\rangle+c'_{+}\langle J'+1,M|\vec{p}\cdot\vec{E}_{L}|J',M\rangle+ \\ c''_{-}\langle J',M|\vec{p}\cdot\vec{E}_{L}|J'-1,M\rangle\text{+}c''_{\text{+}}\langle J',M|\vec{p}\cdot\vec{E}_{L}|J'\text{+}1,M\rangle\\ =E_{s}\left[\alpha'_{-}\langle J'-1,M|\vec{p}\cdot\vec{E}_{L}|J',M\rangle+\alpha'_{+}\langle J'+1,M|\vec{p}\cdot\vec{E}_{L}|J',M\rangle+\right. \\ \left.\alpha''_{-}\langle J',M|\vec{p}\cdot\vec{E}_{L}|J'-1,M\rangle\text{+}\alpha''_{\text{+}}\langle J',M|\vec{p}\cdot\vec{E}_{L}|J'\text{+}1,M\rangle\right] \\ =E_{s}pE_{L}\left[\alpha'_{-}\langle J'-1,M|\cos\theta|J',M\rangle+\alpha'_{+}\langle J'+1,M|\cos\theta|J',M\rangle+\right. \\ \left.\alpha''_{-}\langle J',M|\cos\theta|J'-1,M\rangle\text{+}\alpha''_{\text{+}}\langle J',M|\cos\theta|J'\text{+}1,M\rangle\right] \\ =2E_{s}pE_{L}\left\{\alpha'_{-}\left[\frac{(J'+1)^{2}-M^{2}}{(2J'+1)(2J'+3)}\right]+\alpha'_{+}\left[\frac{(J'+3)^{2}-M^{2}}{(2J'+3)(2J'+5)}\right]\right\} \end{matrix}$ (3)

where $\vec{E}_{s}$ is the static electric field vector measured by the detecting laser electric field $\vec{E}_{L}$. In order to get above formula, the following relations are used

$\alpha'_{-}\approx\alpha''_{-},\alpha'_{+}\approx\alpha''_{-}$,

##

$$\begin{matrix} c'_{-}=\frac{H'_{J',J'-1}}{E_{J',M}-E_{J'-1,M}}\begin{matrix} = & \frac{E_{s}h'_{J',J'-1}}{E_{J',M}-E_{J'-1,M}}=E_{s}\alpha'_{-} & \end{matrix} \\ c'_{+}=\frac{H'_{J',J'+1}}{E_{J',M}-E_{J'+1,M}}\begin{matrix} = & \frac{E_{s}h'_{J',J'+1}}{E_{J',M}-E_{J'+1,M}}=E_{s}\alpha'_{+} & & & & \end{matrix} \end{matrix}$$

##

$$\begin{matrix} \langle J-1,M|\cos\theta|J,M\rangle=\left[\frac{(J+1)^{2}-M^{2}}{(2J+1)(2J+3)}\right] \\ \langle J,M|\cos\theta|J+1,M\rangle=\left[\frac{(J+3)^{2}-M^{2}}{(2J+3)(2J+5)}\right] \end{matrix}$$

## Then the Eq. (3) can be re-written as follows:

## $I_{Q}(r,v_{0})=\sum_{J'=0} I_{Q}(J',v_{0})=\kappa E_{s}^{2}$, (4)

## where $\kappa$absorbs all the constant factors.

## In fact, the numerical simulation (see Fig.3) in main paper gives the quantitative relationship between them in strong electric field. Here we also numerically calculate the dependences of the normalized Q-branch intensity on the electric field intensity of (HCCCN)_3_ in a weak field$E\leq1.0\text{V/cm}$, and the results are shown in Fig.1. We can see from Fig.1 that the fitting formula is a quadratic form $I_{Q}=\kappa E^{2}$, like Eq. (4), but this relation is a little inaccuracy for higher field$E\geq1.0\text{V/cm}$. In order to obtain a higher resolution in weak field, the line width of each possible transition is reduced to 0.00001 cm^-1^ ($\approx$B/300).

Fig.2 shows the same results as like Fig.1, but the rotational temperature is reduced to 0.25K. It is significantly more sensitive than 5K.

**Fig.1 .** The dependences of the normalized Q-branch intensity on the electric field intensity of (HCCCN)_3_ in the field of $E\leq1 0 \text{V/cm}$, the rotational temperature is 5.0 K.

**Fig.2 .** The dependences of the normalized Q-branch intensity on the electric field intensity of (HCCCN)_3_ in the field of $E\leq1 \text{V/cm}$, the rotational temperature is 0.25 K.

1. **Measurement sensitivity**

In the case of a supersonic molecular beam, we assume that the rotational temperature is 5.0 K under an atmospheric pressure condition, which is reasonably in cold molecular experiments [3-6]. At this temperature, most of the molecules are in the electronic ground state. By using supersonic molecular beam theory [7-8], the density of linear diatomic molecules can be given by

## , (7)

which is smaller than the experimental results ($2.8\times10^{15}/cm^{3}$for 0.1% NO/He and $1.2\times10^{15}/cm^{3}$for 0.5% NO/Ar) in Ref.[9]. In Eq.(7), $T_{0}$ is the room temperature and $T$is the rotational temperature of a supersonic molecular beam. In our calculation, we assume that (HCCCN)_3_ is a linear ball molecule, the factor$\gamma$ is chosen as$1.4$.

Because the volume of a supersonic molecular beam is much larger than one of a focused pump light beam, we can determinate the total molecule number interacting with the pumping light only by considering the size of focused laser beam. If the laser wavelength$\lambda=3087.0\text{nm}$,$w_{0}=100\text{μm}$, then $f=\frac{\pi w_{0}^{2}}{\lambda}=0.01051m$, $V_{eff}=4.4024\times10^{-10}m^{3}$and the molecule number in an effective detected volume is given by

## $N_{gs}=nV_{eff}=3.8112\times10^{11}$, (7)

The photon-number signal of Q-branch intensity can be estimated by

## $\begin{matrix} N_{QS}=N_{es}I_{Q} \\ \begin{matrix} & =N_{gs}\rho_{ee} \end{matrix}I_{Q}=\frac{1}{2}nV_{eff}\kappa E^{2} \end{matrix}$ , (8)

where $\rho_{ee}$ is the population of molecule in the excited state, it has a steady-state solution of 0.5 in a two-level Bloch equations when the used resonant laser is at saturation intensity ($s=I/I_{sat}\geq1$)[10].

When without considering the influence of stray light on the LIF detection, and the fluorescence-photon  detecting  noise  is only the shot noise considered in this sensitivity calculation. It has an r.m.s. amplitude of $\sigma_{N_{QS}}=\sqrt{N_{QS}}$. We find that in order to obtain a signal-to-noise-ratio $SNR=N_{QS}/\sigma_{N_{QS}}=\sqrt{N_{QS}}\geq1$, it requires that

## $\eta_{c}nV_{eff}I_{Q}\geq2$, (9)

substituting $I_{Q}$with $\kappa E^{2}$, then the single-shot limited sensitivity of our method to measure static electric field, determined by shot noise coming from the fluctuation of the LIF signal, is given by

## $E(\text{V/cm})=\frac{1}{100}\frac{\sqrt{2}}{\sqrt{\kappa\eta nV_{eff}}}$. (10)

where $\eta_{c}$is the fluorescence collecting efficiency, $n$ is the density of molecules, and $\kappa$the front factor of $E^{2}$. The effective detected volume within probe laser beam can be assumed as a spheroid, it’s [volume](javascript:void(0);) $V_{eff}$ equals to $\frac{4}{3}\pi a^{2}b=\frac{8}{3}\pi w_{0}^{2}f$, $w_{0}$is the waist radius, and $f$represents the  Rayleigh length of the Gaussian beam.

We assume that the fluorescence collecting efficiency of the detected system is 10%, the limited sensitivity of our method can be calculated out. For example, when the rotational temperature of supersonic molecular beam is 5.0 K, the single-shot limited sensitivity will be E=2.05×10^-7^ V/cm. In this case, however, SNR=1, the relative measurement error (or measurement accuracy) is equal to 1/SNR=100%. So in order to obtain a 1% relative measurement error (a relative uncertainty), usually, the SNR should be equal to 100, and the corresponding practical sensitivity is E=2.05×10^-5^ V/cm.

**References:**

[1] Alyabyshev, Sergey V. and Lemeshko, Mikhail and Krems, Roman V. Sensitive imaging of electromagnetic fields with paramagnetic polar molecules. *Phys. Rev. A.* **86**,013409 (2012)

[2] Böhi, Pascal and Riedel, Max F. and Hänsch, Theodor W. and Treutlein, Philipp. Imaging of microwave fields using ultracold atoms. *Appl. Phys. Lett.* **97**, 051101 (2010).

[3] X. Yang, E. R. Th. Kerstel, G. Scoles, R. J. Bemish, and R. E. Miller. High resolution infrared molecular beam spectroscopy of cyanoacetylene clusters. *J. Chem. Phys.* **103**, 8828 (1995).

[4] Congsen Meng, Aernout P. P. van der Poel, Cunfeng Cheng, and Hendrick L. Bethlem. Femtosecond laser detection of Stark-decelerated and trapped methylfluoride molecules. *Phys. Rev. A.* **92**, 023404 (2015).

[5] Block, P. A., Bohac, E. J. & Miller, R. E. Spectroscopy of pendular states: The use of molecular complexes in achieving orientation. *Phys. Rev. Lett.* **68**, 1303-1306 (1992).

[6] K. W. Jucks, and R. E. Miller, Near infrared spectroscopic observation of the linear and cyclic isomers of the hydrogen cyanide trimer, J. Chem Phys., 88, 2196-2204 (1988).

[7] N. F. Ramsey, Molecular Beam, Oxford University Press (1985).

[8] Giovanni Sanna and Giuseppe Tomassetti, Introduction to molecular beams gas dynamics, Imperial College Press (2005).

[9] B. Yan, P. F. H. Claus, B. G. M. van Oorschot, L. Gerritsen, A. T. J. B. Eppink, S. Y. T. van de Meerakker, and D. H. Parker, A new high intensity and short-pulse molecular beam valve, Rev. Scientific instruments, 84, 023102 (2013);

[10] S.Stenholm. Foundations of Laser Spectroscopy, (John Wiley & Sons, Inc., 1984)
